# Multimodal evoked potentials in chronic migraine

**DOI:** 10.1186/1129-2377-14-S1-P122

**Published:** 2013-02-21

**Authors:** G Coppola, E Iacovelli, M Bracaglia, C Di Lorenzo, F Pierelli

**Affiliations:** 1 Sapienza University of Rome Polo Pontino, Department of Medico-Surgical Sciences and Biotechnologies, Latina, Italy; 2 Sapienza University of Rome, Department of medico-surgical sciences and biotechnologies, Neurology section, Rome, Italy

## Background and objectives

Chronic migraine (CM) is a disabling health condition. The exact pathophysiological mechanisms are not completely clarified, but a crucial role was attributed to central sensitization. When still episodic, migraine is characterized by a deficient habituation to any kind of sensorial stimulations between attacks, and by an ictal EPs normalization. Less is known about how central sensitization alter this electrocortical profile in CM. Materials & Method – Fifteen episodic and 14 chronic migraine patients underwent median-nerve somatosensory (SSEPs) (right stimulation, 500 sweeps, 4.4 repetition rate, 1.2 motor threshold) and visual (VEPs) evoked potentials (right eye stimulation, 600 sweeps, 3.1 repetition rate, 15 min of arc check) randomly during the same recording session. Patients groups were compared to a group of 22 healthy volunteers (HV) of comparable age and gender distribution. Habituation was calculated as the slope of the linear regression between block 1 to 3 for SSEPs or between block 1 to 6 for VEPs.

## Results

In episodic migraineurs recorded between attacks, 1st amplitude blocks of VEPs and SSEPs were respectively reduced (p=0.05) or tended to be reduced (p=0.07), but thereafter both failed to habituate along subsequent blocks of responses. In CM patients initial VEP and SSEP amplitudes were in the same range of activation of HV (p>0.05), then habituated normally during stimulus repetitions. When data of MO and CM patients were combined, the SSEP 1st amplitude block was positively (r=0.411, p=0.04) and the slope negatively (r= -0.624, p=0.001) correlated with the monthly number of days with headache.

## Conclusion

Our results show in CM abnormalities that are also reported during attacks in episodic migraineurs, namely response habituation, which contrasts to its lacking detected between attacks. This suggests that from an electrophysiological point of view, CM looks like a never ending migraine attack.

